# DNA Damage and Radiosensitivity in Blood Cells from Subjects Undergoing 45 Days of Isolation and Confinement: An Explorative Study

**DOI:** 10.3390/cimb44020046

**Published:** 2022-01-27

**Authors:** Alan H. Feiveson, Stephanie S. Krieger, Gudrun von Scheven, Brian E. Crucian, Alexander Bürkle, Alexander C. Stahn, Honglu Wu, María Moreno-Villanueva

**Affiliations:** 1 NASA Johnson Space Center, Houston, TX 77058, USA; alan.h.feiveson@nasa.gov (A.H.F.); brian.crucian-1@nasa.gov (B.E.C.); honglu.wu-1@nasa.gov (H.W.); 2 KBR Wyle, Houston, TX 77058, USA; stephanie.s.krieger@nasa.gov; 3 Molecular Toxicology Group, Department of Biology, University of Konstanz, 78457 Konstanz, Germany; gudrun.vonscheven@uni-konstanz.de (G.v.S.); alexander.buerkle@uni-konstanz.de (A.B.); 4 Department of Psychiatry, Perelman School of Medicine, University of Pennsylvania, 1019 Blockley Hall, 423 Guardian Drive, Philadelphia, PA 19104, USA; astahn@pennmedicine.upenn.edu; 5 Center for Space Medicine and Extreme Environments, Institute of Physiology, Charité—Universitätsmedizin Berlin, Corporate Member of Freie Universität Berlin, Humboldt-Universität zu Berlin, 10117 Berlin, Germany; 6 Human Performance Research Centre, Department of Sport Science, University of Konstanz, 78457 Konstanz, Germany

**Keywords:** space environment, stress, DNA damage, radiation

## Abstract

The effect of confined and isolated experience on astronauts’ health is an important factor to consider for future space exploration missions. The more confined and isolated humans are, the more likely they are to develop negative behavioral or cognitive conditions such as a mood decline, sleep disorder, depression, fatigue and/or physiological problems associated with chronic stress. Molecular mediators of chronic stress, such as cytokines, stress hormones or reactive oxygen species (ROS) are known to induce cellular damage including damage to the DNA. In view of the growing evidence of chronic stress-induced DNA damage, we conducted an explorative study and measured DNA strand breaks in 20 healthy adults. The participants were grouped into five teams (missions). Each team was composed of four participants, who spent 45 days in isolation and confinement in NASA’s Human Exploration Research Analog (HERA). Endogenous DNA integrity, ex-vivo radiation-induced DNA damage and the rates of DNA repair were assessed every week. Our results show a high inter-individual variability as well as differences between the missions, which cannot be explained by inter-individual variability alone. The ages and sex of the participants did not appear to influence the results.

## 1. Introduction

There is no doubt that stress caused by long-term isolation and confinement negatively influences our physical and mental health. The health consequences of social isolation have been extensively studied in animal models (for review see [1]). For example, short-term social isolation induced depressive-like behavior, increased corticosterone concentration [2], and caused memory deficits and oxidative stress in mice [3]. In rats, 11 days of isolation stress strongly upregulated plasma interleukin-1β (IL-1β) and the stress hormone adrenocorticotropic hormone (ACTH) levels while corticosterone levels declined [4]. Oxidative stress markers in the hypothalamus were increased in rats as early as two weeks after social isolation [5]. Similar effects have been reported after extended isolation. For instance, an isolation period of 42 days increased glucocorticoid levels, oxidative damage and telomere degradation in prairie voles [6], while individually housed rats for 28 days showed higher levels of adrenocorticotropic hormone (ACTH), as well as in the cytokines tumor necrosis factor alpha (TNF-α), inteleukin-4 (IL-4) and interleukin-10 (IL-10) [7]. The overarching message from these studies is that physical and mental stress induced by isolation can be manifested in altered stress hormones, release of inflammatory molecules and production of reactive oxidative species (ROS), all of which are recognized mediators of DNA damage [8,9,10,11,12,13,14,15,16,17]. Therefore, it is plausible to postulate that isolation stress would induce DNA damage. Indeed, 8-hydroxy-2-deoxyguanosine (8OhdG), a DNA damage marker, has been detected in the hypothalamus of rats isolated for 2 weeks [5].

With the implementation of terrestrial space analogs, the detrimental effects of isolation have also been identified in humans; for example, fatigue, misaligned circadian rhythm, sleep disorders, altered stress hormone levels, and immune modulatory changes have been noted [18]. A recent systematic meta-analysis reveals an association between loneliness, social isolation and C-reactive protein (CRP), fibrinogen and Interleukin-6 (IL-6), thus suggesting a possible link between social isolation and loneliness with systemic inflammation [19]. Granulocyte-mediated H_2_O_2_ production and shedding of the cell adhesion molecule CD62L were increased in six men confined for 105 days to a simulated space module of ~500 m^3^ [20]. Healthy humans confined in a chamber for 520 days presented signs of behavioral disturbances and psychological distress [21], decreased cortical activity, increased salivary cortisol [22,23,24], increased lymphocyte count and heightened immune responses [24]. In a study of men isolated during the Antarctic winter, anti-inflammatory cytokines (IL-10 and IL-1RA) decreased whereas proinflammatory IFN-γ increased, suggesting that long term isolation of human beings could shift the plasma cytokine balance towards a proinflammatory profile [25].

Results from studies of humans confined in the NASA underwater habitat NEEMO suggested that short-term (10- to 15-d) confinement and isolation leads to oxidative stress, including DNA oxidation, and changes in biomarkers of DNA damage and repair [26,27,28]. However, NEEMO is a very special setting with hyperbaric hyperoxic conditions, which act as a confounder [18]. Therefore, for our present study of DNA damage we utilized NASA’s human exploration research analog (HERA), which supports isolation and confinement of humans under normal atmospheric conditions. In general, HERA was built to allow the investigation of reaction to stress, signs of early depression, anxiety and anger and their impact on human performance and health as well as biopsychosocial adaptation under confinement.

There is extensive evidence of stress-induced DNA damage mediated by cytokines [8,13,15], ROS [9,10,11] and stress hormones [12,14,16,17]. However, to our knowledge, how social isolation affects DNA integrity in humans has not been explicitly investigated. Explorative studies can provide vital information to support more robust evaluations and are a key step in assessing the feasibility and value of progressing to an effectiveness study [29]. Therefore, we conducted an explorative study assessing the DNA integrity in immune cells from individuals of the HERA cohort. In our study, we detected changes in endogenous DNA integrity, ex vivo radiation-induced DNA damage and DNA repair in volunteers undergoing 45 days of isolation and confinement in the HERA facility. We also provide possible explanations for the observed effects that can help to design future research.

## 2. Results

We observed a high inter-individual variability leading to no significant effects when including all subjects of all missions in the analyses. Interestedly, the inter-individual variability is considerably reduced within the mission. Therefore, the effects of confinement on DNAI and ADRR are analyzed and plotted for each mission separately. 

### 2.1. Effect of Confinement on DNAI before Irradiation

**Figure 1:**
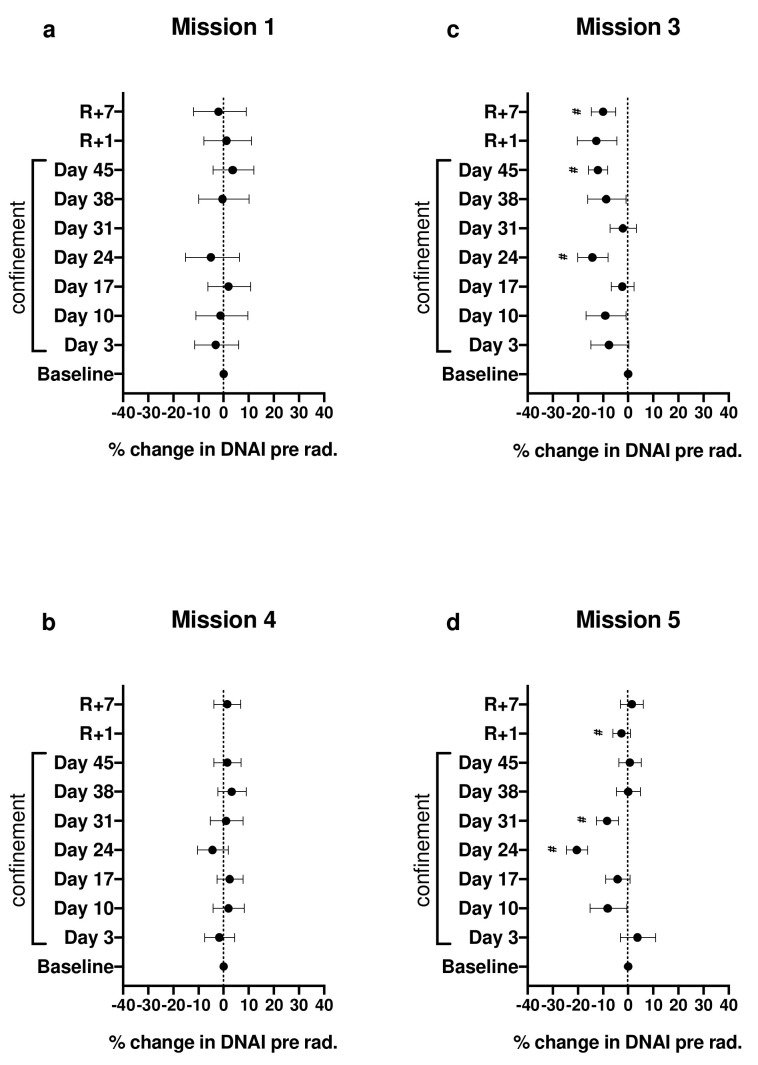
Percent changes in DNAI pre-irradiation (pre rad.) median relative to DNAI baseline (day C-10); # denotes adjusted *p* < 0.1 for false-discovery rates (FDRs). Error bars represent 95% CI. (**a**–**d**) are missions 1, 4, 3 and 5 respectively.

The overall effect of confinement varied considerably between missions (χ^2^(28) = 167; *p* < 10^−20^). No significant changes were observed in the DNA integrity (DNAI) in blood cells from subjects during missions 1 and 4 (Figure 1a,b), while it changed significantly during missions 3 and 5 (Figure 1c,d). The estimated median DNAI was between 2% and 14% lower than baseline for all confinement and both recovery sessions of mission 3 (Figure 1c), with the most notable reductions being 14% for day 24 (95% conf = (−20%, −8%; adj *p* = 0.0007)), for 12% for day 45 (95% conf = (−16%, −8%; adj *p* < 10^−6^)), and 10% for recovery day 7 (95% conf = (−15%, −5%; adj *p* = 0.005)). For mission 5 (Figure 1d), a large loss in median DNAI (20%) was also observed on day 24 (95% conf = (−24%, −16%; adj *p* < 10^−14^)) and on day 31 (8%) (95% conf = (−13%, −4%; adj *p* = 0.012)). Unlike the case for mission 3, estimates of median DNAI for mission 5 were generally lower than baseline only up to day 31, but not afterwards. 

### 2.2. Effect of Irradiation on DNAI during Confinement

**Figure 2:**
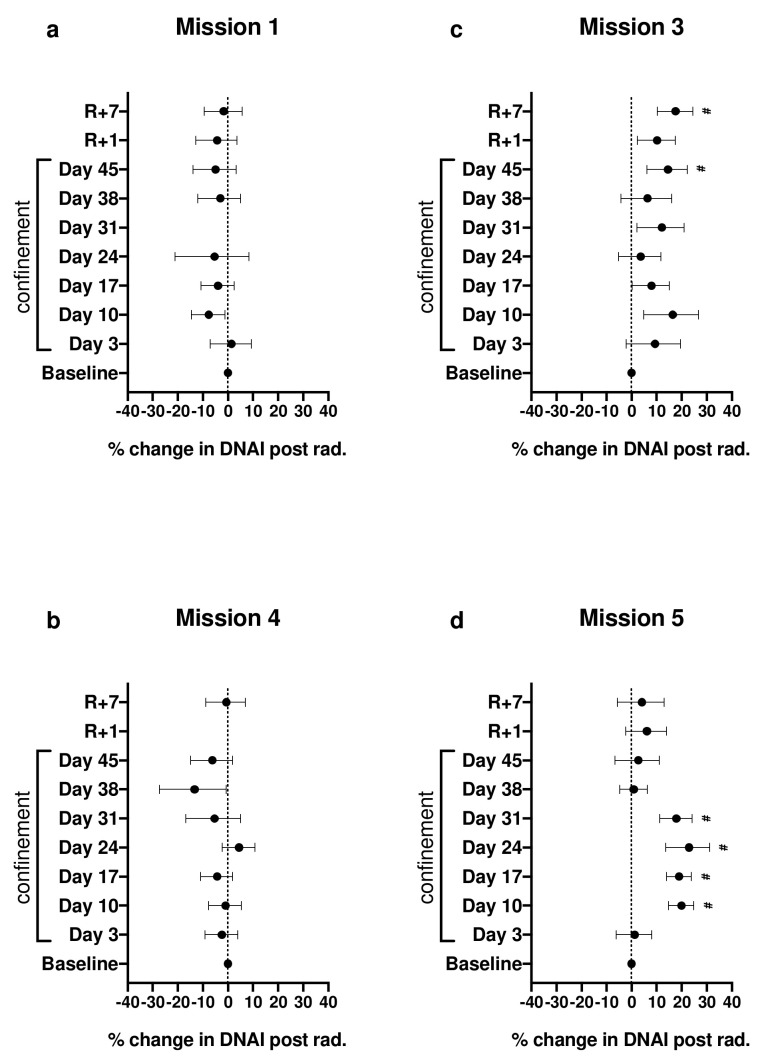
Percent difference between irradiation (post rad.) effect on DNAI median at each session relative to irradiation effect at baseline (day C-10). Irradiation effect is defined as the ratio of median DNAI post irradiation to DNAI median pre-irradiation. # denotes adjusted *p* < 0.1 for false-discovery rates (FDRs). Error bars represent 95% CI. (**a**–**d**) are missions 1, 4, 3 and 5 respectively.

Immediately after irradiation, median DNAI was reduced by about 40% ± 2.4% (1SE) for samples obtained at the baseline session when compared to non-irradiated samples. As was the case for DNAI before irradiation, the effect of confinement on the response to irradiation varied considerably between missions (χ^2^(28) = 245; *p* < 10^−35^). Here again, no significant differences were observed in the DNAI response to irradiation in blood cells from subjects during missions 1 and 4 (Figure 2a,b), while it changed significantly during missions 3 and 5 (Figure 2c,d). The most obvious exacerbation to the effect of irradiation on DNAI occurred on days 10 through 31 of mission 5, where the reduction in median DNAI after irradiation was about 20% higher than on the baseline day. We also observed a general pattern of an exacerbated effect of irradiation throughout mission 3, even 7 days after recovery, the effect of irradiation was still about 17% higher than at baseline (adjusted *p* = 0.0004) (Supplementary Table S1).

### 2.3. Effect of Confinement on DNA Repair Rate

**Figure 3:**
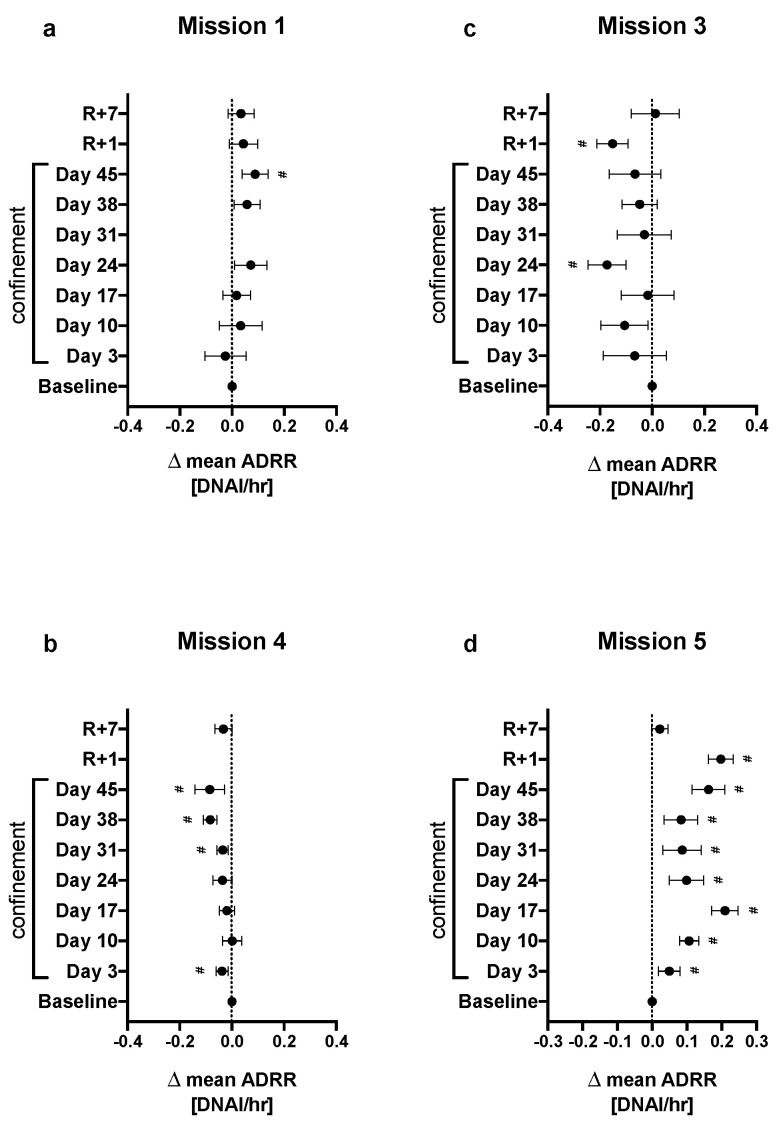
Difference in mean ADRR (DNAI/hr) between each session and baseline ADRR (day C 10). # denotes adj *p* < 0.1 for false-discovery rates (FDRs). Error bars represent 95% CI. (**a**–**d**) are missions 1, 4, 3 and 5 respectively.

The effect of confinement on mean average DNAI repair rate (ADRR) varied considerably between missions. In missions 1 and 5 (Figure 3a,d) the mean ADRR (DNAI/hr) was higher than at baseline, whereas the opposite effect was observed in missions 3 and 4 (Figure 3b,c), in which the mean ADRR was significantly lower (Supplementary Table S2).

### 2.4. Effect of Age and Sex

Ages of subjects varied between 29 and 55 years; however, there is no evidence of age affecting DNAI either before or after irradiation (*p* = 0.82). With only 20 subjects (13 male, 7 female) we did not observe conclusive evidence of an overall sex difference in median DNAI (*p* = 0.07). While the 95% confidence interval for median DNAI (−4.4%, +0.2%) suggests a possible overall lower DNAI for females versus males, there was no evidence of a differential sex effect on DNAI with respect to irradiation (*p* = 0.97) (or any of the days in HERA (χ^2^(9) = 9.4; *p* = 0.40). There was also no evidence of an overall effect on mean DARR either with sex (*p* = 0.67) or with age (*p* = 0.83), nor of any interaction between sex (*p* = 0.91) or age (*p* = 0.95).

## 3. Discussion

Animal models have produced a significant contribution to our understanding of the effects of several kinds of stress on the human body [30]. However, studies addressing the effect of confinement- and isolation-induced stress on DNA integrity in healthy humans has not been reported so far. Analogs used in spaceflight research represent an opportunity to investigate the effects of confinement and isolation in humans. HERA provides a unique controlled environment to investigate the effects of prolonged isolation as well as the body’s adaptation and resilience to isolation, confinement and social stress. 

Including all subjects from all missions in the analyses, our results show a high inter-individual variability. This is not surprising, considering data from several decades of molecular epidemiology indicating that DNA repair capacity varies significantly among individuals (for an extensive review see [31]). However, the inter-individual variability decreased when analyzing the subjects grouped by missions. We found significant differences in DNAI during the time of confinement in missions 3 and 5 but not in missions 1 and 4. This gives rise to the question of team composition. Interpersonal issues have been consistently identified as substantial sources of stress during spaceflight and in Isolated, Confined and Extreme (ICE) environments [32,33,34,35]. However, subject selection prioritized individuals considered to be “astronaut-like” in terms of technical skill, educational status, military experience and age range [36]. Individuals of a high-performance working team should have complementary skills to cope with stress and adapt to difficult environments. Although, the possibility of higher stress due to interpersonal issues cannot be excluded, the crew selection criteria should reduce this risk.

Another highly interesting point to mention is the diet. In this study, subjects of two missions were on a shelf-stable spaceflight “standard” diet and subjects of the two other missions were on an “enhanced” shelf-stable spaceflight diet with 25% more foods rich in omega-3 fatty acids, lycopene, flavonoids and more fruits, and vegetables in general [37]. Although we do not know which team was on which diet, considering this possibility for future analyses is entirely plausible since the protective effect of micronutrients and antioxidants on DNA integrity has been extensively reported. Several essential nutrients are required for the prevention of DNA oxidation (vitamin C, vitamin E, zinc, manganese, selenium), and DNA damage sensing and repair (niacin, zinc, iron, magnesium). Deficiency in these micronutrients increases genomic instability and the susceptibility to DNA damage caused by endogenous and environmental stressors [38]. These observations have led to the emerging science of genome health nutrigenomics, which is based on the principle that DNA damage could be nutritionally prevented [39]. Furthermore, the DNA damage and repair trial—the DART study—investigated the inter-individual variation in DNA damage, the capacity for base excision repair (BER) and the responses of both variables to supplementation with antioxidants for 6 weeks in 48 volunteers. The authors reported a high inter-individual variation in endogenous lymphocyte DNA strand breaks (8-fold variation), in damage after a challenge with H_2_O_2_ (16-fold variation) and in DNA repair (41-fold variation). Pre-supplementation significantly reduced the level of endogenous DNA damage but there was no effect of supplementation on BER [40], indicating that the BER pathway might not be affected by diet. Our results indicate an impaired DNA repair rate after radiation in all missions, which also suggest that these changes might not depend on diet. Genetic factors could also explain, at least in part, the individual differences in DNA repair rate. Indeed, several amino acid variants have been identified in DNA repair genes [41,42,43]. However, in all four completed missions, the DNA repair rate did not differ from baseline 7 days after egress suggesting a confinement (environmental)-associated effect rather than a genotypic effect. On the other hand, the impact of genetic variation might become “visible” under confinement conditions. Interestingly, missions 1 and 5 showed a “faster”, while mission 3 and 4 a “slower”, DNA repair rate. Therefore, the question of team composition arises again. 

Our results also show a decrease in radiation-induced DNA damage in missions 3 and 5 but not in 1 and 4. Paradoxically, missions 3 and 5 showed a higher endogenous DNA damage and one would expect these cells to be more radiosensitive. However, cells exposed to chronical sublethal stress might develop adaptive responses that enhance the ability of the cells to cope with more severe and acute stress such as radiation. There is evidence that an adaptive response to chronic, low-level oxidative stress results in genomic protection and up-regulated maintenance of cellular homeostasis [44]. Interestingly, DNA single strand breaks induced through prolonged oxidative stress trigger cell survival in response to genotoxic stress under nutrient restriction [45].

Although our data generally indicate an impaired DNA damage response in subjects undergoing confinement, efforts at making definitive conclusions from this study are compromised due to the limited number of samples that can be analyzed at once and the fact that the missions were scheduled at different times of the year, thus creating unavoidable confounding effects of missions and time of measurements. Furthermore, uncertainties in estimates of median DNAI and ADRR were large because of the small number of subjects taking part in each mission, as well as the relatively high variability between missions. In particular, the etiology of differences in DNA damage between days within a mission are difficult to assess with this limited amount of information. 

## 4. Materials and Methods

### 4.1. Subjects

The NASA Human Research Program Human Exploration Research Analog (HERA) is a high-fidelity space analog isolation facility located at the Johnson Space Center in Houston, TX, USA. The total space comprises 148.6 m^3^ distributed as follows: core 56.0 m^3^, loft 69.9 m^3^, airlock 8.6 m^3^, and hygiene module 14.1 m^3^. Capabilities, standardized conditions and study requirements are specified in the Research Operations and Integration (ROI) document published by the NASA Human Research Program [36] Twenty healthy men and women participated in this study. Informed consent was obtained from all participants. Five teams of four subjects each participated in 45-day “missions” (missions 1–5) in isolation inside the HERA facility, with the exception of the four participants in mission 2 who were required to leave the facility at day 17 due to extreme weather conditions in the Houston area (Hurricane Harvey 2017). 

Participants were recruited and initially screened by the National Aeronautics and Space Administration (NASA) at the Johnson Space Center. Participants must pass a modified Class III flight physical. They must demonstrate technical skills. They must have demonstrated motivation and a work ethic similar to the current astronaut population. Psychological assessment by a clinical psychologist is necessary to qualify for participation. Astronaut-like characteristics that are considered during HERA test subject selection include a bachelor’s degree from an accredited institution in engineering, biological science, physical science, or mathematics. An advanced degree (e.g., M.S.) in STEM field is preferred and may be substituted for experience as follows: master’s degree = 1 year of experience, Doctoral degree = 3 years of experience. Military experience may be considered equivalent years of experience [36]. Only healthy subjects with no history of cardiovascular, neurological, gastrointestinal, or musculoskeletal problems were selected. On each of ten selected mission time points (days C-10, 3, 10, 17, 24, 31, 38, 45, R + 1, and R + 7, where “C-10” denotes 10 days prior to confinement, “R + 1” denotes 1 day after recovery, and “R + 7” denotes 7 days after recovery), blood was drawn in the morning after an overnight fast. The study was approved by the Institutional Review Board of NASA (NASA 7116301606HR) and was carried out in accordance with the Declaration of Helsinki.

### 4.2. Blood Draw and Sample Processing

Venous blood was obtained using BD Vacutainer^®®^ Acid Citrate Dextrose (ACD)-Glass tubes (BD Biosciences, Franklin Lakes, NJ, USA). Peripheral blood mononuclear cells (PBMCs) were isolated from whole blood by Ficoll-PaqueTM PLUS (GE Healthcare, Uppsala, Sweden) density gradient centrifugation following the manufacturer’s instructions. Isolated PBMCs were transferred into 15-mL tubes and washed with phosphate buffered saline (PBS) (Gibco^®®^, Waltham, MA, USA). Tubes were then centrifuged at 300× *g* for 10 min, the supernatant was removed, and the cell pellet was resuspended in TexMacs medium (Miltenyl Biotec, Auburn, CA, USA). Cells were counted using Guava ViaCount technology (EMD Millipore Co., Hayward, CA, USA). Isolated cells were suspended in 1 mL of freezing medium containing 20% Roswell Park Memorial Institute medium (RPMI-1640) medium, 10% dimethyl sulfoxide (DMSO), and 70% fetal calf serum (FCS), and stored overnight at −80 °C in a Mr. Frosty™ Freezing Container (Thermo Fisher Scientific, Waltham, MA, USA). The cells were then transferred to a liquid nitrogen tank at −180 °C until shipment to Konstanz (Germany), where cells were kept at −196 °C until analysis. 

### 4.3. Cell Thawing Procedure

PBMCs were carefully thawed by immersing cryovials in a water bath at 37 °C until a small amount of ice remained in the cryovial. Cells were transferred to a polypropylene 15 mL tube and a thawing medium (90% RPMI and 10% FCS) was gradually added (1 mL, 1 min later additional 2 mL, 1 min later additional 4 mL). The tube was centrifuged at 300× *g* for 10 min. The cell pellet was gently resuspended in 1.3 mL RPMI medium supplemented with 1% penicillin/streptomycin (without phenol red, without FCS), wherein the cell concentration and viability (determined by electric current exclusion) were assessed using CASY cell counter technology (Innovatis, Zürich, Switzerland). 

### 4.4. Induction of DNA Strand Breaks through Irradiation

Cells (4 × 10^6^/mL) in suspension buffer (0.25 M meso-inositol; 10 mM sodium phosphate, pH 7.4; 1 mM magnesium chloride) were irradiated (Biological X-ray Irradiator X-RAD 225 iX from Precision X-Ray, Inc., North Branford, CT, USA) on ice for 380 s at a dose rate of 0.59 Gy/min (70 kV, 30 mA, 70 cm distance, 1.25 mm Al filter) resulting in a total dose of 3.73 Gy. Radiation-induced DNA strand breaks were assessed immediately after irradiation. In order to track repair of DNA strand breaks through time, cells were incubated at 37 °C for either 10, 20, 30, 40, and 50 min. 

### 4.5. Detection of DNA Strand Breaks

Both prior to and after radiation, cells were transferred to a 96-well plate using a liquid handling device. Within each well, DNA strand breaks were detected using the automated version of the “Fluorimetric detection of Alkaline DNA Unwinding” (FADU) assay [46,47]. This assay is based on controlled (time, pH, and temperature) DNA unwinding. Double-stranded DNA is then detected by fluorescence using the specific dye SybrGreen^®®^ (ThermoFisher, Göttingen, Germany). More specifically, the higher the florescence signal, the higher the amount of DNA without strand breaks. In order to determine the total amount of intact double-stranded DNA in a cell lysate, the unwinding of DNA was prevented by first adding a buffer to neutralize the effect of the alkaline unwinding solution. The resulting fluorescence signal was taken as a reference value assumed to reflect 100% of the original DNA amount. For each experimental condition (Supplementary Table S3), after reversing the order of alkaline treatment and neutralization, a decrease in the fluorescence intensity indicates an increase of DNA unwinding and, consequently, a higher number of DNA strand breaks. DNA integrity (DNAI) was then defined by the ratio of the fluorescence signal measured from the experimental sample to the corresponding reference value.

### 4.6. Experimental Design

For each of the 20 subjects, the original experimental design called for 28 measuring points (seven irradiation conditions × four replicates) at each of the ten mission time points (Supplementary Table S3). Additionally, four samples (one for each replicate) at each of the ten mission time points served as reference values for maximal DNAI, making a total of 320 measuring points per subject. However, due to uncontrollable weather events, technical failures and missing blood samples, data were obtained at all ten mission time points for only eight subjects, at nine time points for an additional eight subjects, and at four time points for the remaining four subjects. 

### 4.7. Data Analysis

Separate mixed-model regression analyses were performed, one with log DNAI as the dependent variable and another with average repair rate (DNAI/minute) as the dependent variable. Mixed-model residuals under the log transformation of DNAI closely conformed to model assumptions of normality as did residuals with an untransformed average repair rate (ADRR). Differences in means of log DNAI can be expressed as ratios of median DNAI, whereas differences in mean ADRR can be estimated directly from the fitted regression model. ADRR was estimated from a cubic-spline fit to DNAI time-response data after irradiation. More specifically, these regressions were used to estimate the effects of days within the missions, missions, and irradiation while adjusting for missing data as well as random differences between subjects and batches. Although the limited amount of available data from mission 2 was used in the regression analyses, there were not enough of these data to allow reporting of results for this mission. In addition, modifications to the original regression analysis models were made to investigate the possible effects of age and sex. Error bars represent 95% CI. Mathematical details of these statistical models along with examples showing how values in Results tables were obtained are given in Appendix A.

For all regressions, standard errors were estimated by bootstrapping with 200 replicates, implemented with the “bootstrap” and “mixed” commands in Stata16 statistical software StataCorp. 2019. Stata Statistical Software: Release 16. College Station, TX, USA: Stata Corp LP. For all outcomes, *p*-values for testing the effect of each confinement session or post-confinement session relative to their own baseline were adjusted upwards to reflect false-discovery rates (FDRs) for multiple testing of 111 null hypotheses (37 for pre-irradiation, 37 for post-irradiation, and 37 for DNAI repair rate scenarios). In the reporting of results, an FDR of 0.10 or less was used as a basis for flagging interesting session effects in this exploratory study. FDR values, denoted by “adj *p,*” were computed using the qqvalue command in Stata 16 software [48].

## 5. Conclusions

Despite the above limitations, we nevertheless observed substantial evidence of change in the level of DNA integrity in some of the five groups of subjects undergoing confinement and isolation. Further scrutiny of the four HERA mission parameters for which we had fairly complete data resulted in indications of possible team-composition and/or diet-associated effects on DNAI. Other factors that could have affected the DNAI and DNA repair rate in the HERA subjects are stress level, exercise habits and sleep quality/amount. Conjectures such as those stated above could be better assessed with a larger controlled study of team reaction to stress, whether it be due to confinement or other situational environments.

## Figures and Tables

**Figure A1:**
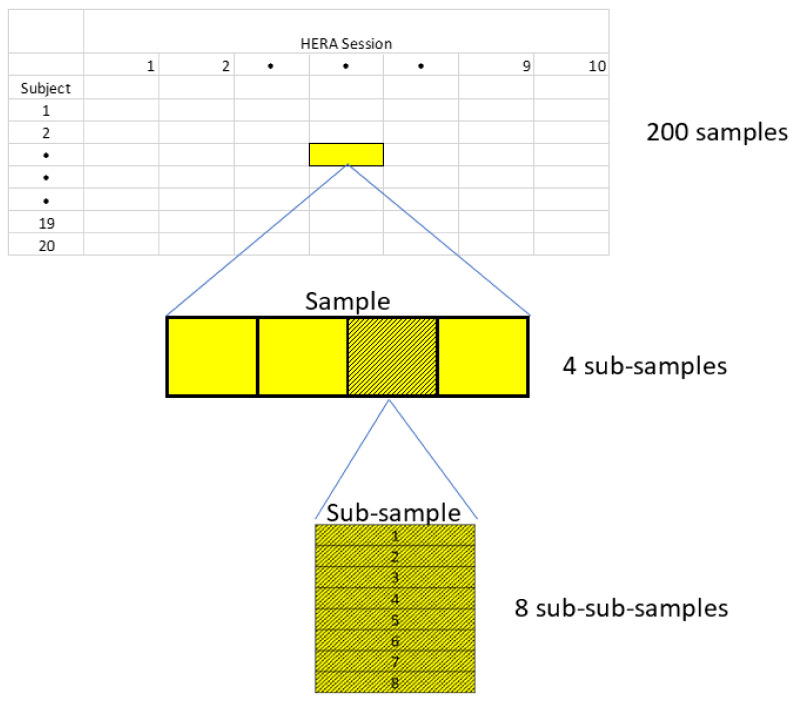
Depiction of Experimental Design.

## Data Availability

Mathematical details of these statistical models along with examples showing how values in results tables were obtained are given in the Appendix A.

## Supplementary Materials

The following supporting information can be downloaded at: https://www.mdpi.com/article/10.3390/cimb44020046/s1, Table S1: Percent reduction in median DNAI after irradiation relative to reduction at baseline, along with 95% confidence intervals and adjusted *p* values Table S2: Change in mean ADRR (DNAI/hr), 95% confidence intervals, and adjusted *p*-values; Table S3: Allocation of samples to 7 experimental conditions for a given subject and mission time point as originally planned.

## Appendix A

This supplement contains a detailed description of the experimental design of this Study, upon which the statistical models for the observed data were based. It also includes a detailed description of the models themselves and how they were used to produce the results reported in the main manuscript.

### Appendix A.1. Experimental Design (as Originally Planned)

- 20 subjects
- 10 HERA sessions (Table A1)
- Separate sample of lymphocytes for each combination of subject and session = 200 samples total
- 4 subsamples for each sample (“replicates”)
- 8 sub-subsamples (7 experimental conditions plus one viability assessment) (Table A2, Figure A1)

Each subsample is divided into 8 sub-subsamples (one for each experimental condition plus one for reference value assessment). This makes a total of 32 sub-subsamples taken from each of the 200 samples, resulting in 6400 sub-subsamples of leukocytes for potential processing (Figure A1).

- 100 fluorescence microplates

Sub-samples were originally planned to be allocated to fluorescence microplates in a partially balanced design with 64 subsamples per plate (Table A3)

**Table A1:** HERA Sessions (=mission days).

HERA Session	Day
1	pre-confinement
2	Day 3
3	Day 10
4	Day 17
5	Day 24
6	Day 31
7	Day 38
8	Day 45
9	Recovery Day 1
10	Recovery Day 7

**Table A2:** Sub-subsample characteristics–Viability assessment and 7 experimental conditions for assessing DNAI. Irradiation was for 380 sec. at a dose rate of 0.59 Gy/min. Total dose = 3.73 Gy.

Sub-Subsample	Exp Condition	X-ray Radiation	Incubation Time (min)
1	Viability	none	none
2	Pre-irradiation	none	none
3	Time Point 1	3.73 Gy	0
4	Time Point 2	3.73 Gy	15
5	Time Point 3	3.73 Gy	30
6	Time Point 4	3.73 Gy	45
7	Time Point 5	3.73 Gy	60
8	Time Point 6	3.73 Gy	75

**Table A3:** Allocation of sub-samples to fluorescence microplates.

Plate	Subjects	Mission	Session	# of Sub-Samples
1	1,2	1	Baseline	64 *
2	3,4	1	Baseline	64
3	5,6	2	Baseline	64
4	7,8	2	Baseline	64
5	9,10	3	Baseline	64
6	11,12	3	Baseline	64
7	13,14	4	Baseline	64
8	15,16	4	Baseline	64
9	17,18	5	Baseline	64
10	19,20	5	Baseline	64
11	1,2	1	Day 3	64
12	3,4	1	Day 3	64
13	5,6	2	Day 3	64
14	7,8	2	Day 3	64
15	9,10	3	Day 3	64
16	11,12	3	Day 3	64
17	13,14	4	Day 3	64
18	15,16	4	Day 3	64
19	17,18	5	Day 3	64
20	19,20	5	Day 3	64
Plates 21–30: same as above, except session is Day 10				
Plates 31–40: same as above, except session is Day 17				
Plates 41–50: same as above, except session is Day 24				
Plates 51–60: same as above, except session is Day 31				
Plates 61–70: same as above, except session is Day 38				
Plates 71–80: same as above, except session is Day 45				
Plates 81–90: same as above, except session is R + 1				
Plates 91–100: same as above, except session is R + 7				

* 4 reps × 8 experimental conditions × 2 subjects = 64 subsamples.

### Appendix A.2. Missing Samples/Data

The realized allocation reflects the failure to collect samples or usable data for certain combinations of missions and sessions:

- The 4 participants (subjects 5–8) in the second mission were required to leave the HERA facility at day 17 due to extreme weather conditions in the Houston area (Hurricane Harvey 2017).
- No data was gathered for day 31 of Mission 1 (Subjects 1–4) because of technical problems with the fluorescence measurements.
- Blood samples were not gathered in the HERA on day R + 1 of Mission 4 (Subjects 13–16).

As a result, 5376 of 6400 (84%) planned observations were available for data analysis.

### Appendix A.3. DNAI Mixed-Model Regression Analysis

For this analysis, the dependent variable was DNAI measured before irradiation and immediately after irradiation (experimental conditions 2 and 3, Table A2). Let $y\equiv y\left(m,i,k,v,j,r\right)\;$ denote DNAI observed for subject *i* in HERA mission *m*, during HERA session *k* based on the *r*-th replicate of fluorescence measurements obtained from the *v*-th microplate under experimental condition *j*. Then in the mixed-model regression analysis for this study, logy was represented as

(A1) $$\log y=\mu +\alpha_{m}+\beta_{k}+{\left(\alpha \beta \right)}_{mk}+P_{j}+{\left(P\alpha \right)}_{mj}+{\left(P\beta \right)}_{kj}+{\left(\alpha \beta P\right)}_{mkj}+u_{i}+h_{v}+e$$

where

*µ* = mean DNAI for the HERA protocol associated with mission 5 prior to confinement and prior to irradiation.

$\alpha_{m}$ = mean effect of the *m*-th mission relative to mission 5. (Note: Mission 5 is treated as the “baseline” mission, hence $\alpha_{5}=0$, by definition. The choice of which mission is baseline is arbitrary and does not affect the results.)

$\beta_{k}$ = mean effect of the *m*-th HERA session relative to the baseline session.

${\left(\alpha \beta \right)}_{mk}$ = interaction effect between the *m*-th mission and *k*-th HERA session.

$P_{j}$ is the indicator for irradiation ($P_{j}$ = 0 pre-irradiation and $P_{j}$ = 1 for post-irradiation)

${\left(P\alpha \right)}_{mj}$ = interaction between the effect of the *m*-th mission and irradiation.

${\left(P\beta \right)}_{kj}$ = interaction between the effect of the *k*-th session and irradiation.

${\left(\alpha \beta P\right)}_{mkj}$ = 3-way interaction between the effects of the *m*-th mission, *k*-th session and irradiation.

$u_{i}$ = random perturbation associated with the *i*-th subject.

$h_{v}$ = random perturbation associated with the *v*-th fluorescence microplate.

e = random error term representing the effects of replicates and all interactions involving subjects and plates.

### Appendix A.4. Calculation of Plotted Values Shown in Figures 1 and 2 of the Primary Article

Under the assumption that the distribution of log *y* is symmetric, exponentiated differences in E(logy) become ratios of median *y*. In particular, for session *k* and mission *m*,

$$\frac{med(y|k,m)}{med(y|baseline,m)}=\exp\{E(\log y|k,m)-E(\log y|baseline,m)\},$$

which in terms of the model parameters in Equation (A1) becomes

(A2) $$\frac{med(y|k,m)}{med(y|baseline,m)}=\exp\{\beta_{k}+{\left(\alpha \beta \right)}_{mk}\}\;\;$$

These ratios, calculated from estimates of the model parameters, were then converted to percent changes (i.e., $\%\;change=100\times \left(ratio-1\right),\;$ which are the point estimates shown in Figure 1. Confidence limits in Figure 1 were obtained by exponentiation of corresponding confidence limits for $\beta_{k}+{\left(\alpha \beta \right)}_{mk}$ and subsequent conversion to percent changes.

Similarly, the reduction in median DNAI after irradiation relative to the reduction at baseline is $\exp\{{∆}^{2}E(\log y)\}$, where for session *k* and mission *m*, ${∆}^{2}E(\log y)$ is the 2nd difference

(A3) $$\left[E\left(\log\;y\;|\mathrm{post}-\mathrm{irradiation},k,m\right)-E\left(\log\;y\;|\mathrm{pre}-\mathrm{irradiation},k,m\right)\right]-\left[E\left(\log\;y\;|\mathrm{post}-\mathrm{irradiation},\mathrm{baseline},m\right)-E\left(\log\;y\;|\mathrm{pre}-\mathrm{irradiation},\mathrm{baseline},m\right)\right]$$

In terms of the model (Equation (A1)), Equation (A3) reduces to

(A4) $${∆}^{2}E(\log y)={\left(P\beta \right)}_{k1}+{\left(\alpha \beta P\right)}_{mk1}$$

Point estimates and confidence limits for the percent change in the irradiation effect shown in Figure 2 and Table S2 were obtained after substituting parameter estimates into Equation (A4) followed by exponentiation and conversion to percent change as above.

The command used in Stata 16 software to fit the model (Equation (A1)) with *zy* = log *y* as the dependent variable and with standard errors of parameter estimates obtained from 200 bootstrap samples is shown below:

bootstrap _b, strata(isess) cluster(id) reps(200) seed(7777777) dots(10) : mixed zy i.isess##i.mission i.isess##i.post##b5.mission ||_all: R.id ||_all: R.plate ,reml

(running mixed on estimation sample)

### Appendix A.5. Mixed-Model Regression Analysis for Average DNAI Repair Rate (ADRR)

For this analysis, the dependent variable was the average DNAI repair rate (ADRR) in units of DNAI/hour calculated from cubic spline fits to the DNAI repair data after irradiation for every combination of subject and session.

For a given subject and HERA confinement session, let $y_{rt}$ be the observed DNAI obtained from replicate *r* at time *t* minutes after irradiation and let $\hat{y}\left(t\right)$ be the spline fit to the values $\left\{y_{rt}\right\}$ as a function of t. Then the average rate of DNAI repair (ADRR) in units of DNAI repair per hour was calculated as

$$ADRR=60\frac{\hat{y}\left(75\right)-\hat{y}\left(0\right)}{75}$$

Let $A\equiv ADRR\left(m,i,k,v\right)\;$ denote the ADRR calculated from DNAI repair data observed for the *i*-th subject during session *k* of HERA mission *m*, based on fluorescence measurements obtained from the *v*-th microplate. Then in the mixed-model regression analysis for this outcome variable, A was represented by

(A5) $$A=\mu +\alpha_{m}+\beta_{k}+{\left(\alpha \beta \right)}_{mk}+h_{v}+e$$

where

μ = mean ADRR for the HERA protocol associated with mission 5 prior to confinement.

$\alpha_{m}$ = mean effect of the *m*-th mission relative to mission 5. (Note: Mission 5 is treated as the “baseline” mission, hence $\alpha_{5}=0$, by definition. The choice of which mission is baseline is arbitrary and does not affect the results.)

$\beta_{k}$ = mean effect of the *m*-th HERA session relative to the baseline session.

${\left(\alpha \beta \right)}_{mk}$ = interaction effect between the *m*-th mission and *k*-th HERA session.

$h_{v}$ = random perturbation associated with the *v*-th fluorescence microplate.

e = random error term representing the effects of replicates and all interactions involving subjects and plates.

### Appendix A.6. Calculation of Plotted Values Shown in Figure 3 of the Primary Article

The effect of confinement on mean ADRR for mission *m* and session *k* is

(A6) $$E\left(A|m,k\right)-E\left(A|m,\;baseline\right)=\beta_{k}+{\left(\alpha \beta \right)}_{mk}$$

Values of the estimated confinement effects plotted in Figure 3 were obtained after substitution of parameter estimates for actual parameters in Equation (A6).

The command used in Stata 16 software to fit the model given by Equation (A5) with standard errors of parameter estimates obtained from 200 bootstrap samples is shown below:

. bootstrap _b, strata(isess) cluster(id) reps(200) seed(7777777) dots(10) : mixed A i.isess##b5.mission ||plate: ,reml

## References

[1] Mumtaz F., Khan M.I., Zubair M., Dehpour A.R. (2018). Neurobiology and consequences of social isolation stress in animal model-A comprehensive review. Biomed Pharm..

[2] Takatsu-Coleman A.L., Patti C.L., Zanin K.A., Zager A., Carvalho R.C., Borcoi A.R., Ceccon L.M., Berro L.F., Tufik S., Andersen M.L. (2013). Short-term social isolation induces depressive-like behaviour and reinstates the retrieval of an aversive task: Mood-congruent memory in male mice?. J. Psychiatry Neurosci..

[3] Famitafreshi H., Karimian M. (2018). Assessment of Improvement in Oxidative Stress Indices with Resocialization in Memory Retrieval in Y-Maze in Male Rats. J. Exp. Neurosci..

[4] Gadek-Michalska A., Tadeusz J., Bugajski A., Bugajski J. (2019). Chronic Isolation Stress Affects Subsequent Crowding Stress-Induced Brain Nitric Oxide Synthase (NOS) Isoforms and Hypothalamic-Pituitary-Adrenal (HPA) Axis Responses. Neurotox. Res..

[5] Colaianna M., Schiavone S., Zotti M., Tucci P., Morgese M.G., Backdahl L., Holmdahl R., Krause K.H., Cuomo V., Trabace L. (2013). Neuroendocrine profile in a rat model of psychosocial stress: Relation to oxidative stress. Antioxid. Redox Signal..

[6] Stevenson J.R., McMahon E.K., Boner W., Haussmann M.F. (2019). Oxytocin administration prevents cellular aging caused by social isolation. Psychoneuroendocrinology.

[7] Krugel U., Fischer J., Bauer K., Sack U., Himmerich H. (2014). The impact of social isolation on immunological parameters in rats. Arch. Toxicol..

[8] Aivaliotis I.L., Pateras I.S., Papaioannou M., Glytsou C., Kontzoglou K., Johnson E.O., Zoumpourlis V. (2012). How do cytokines trigger genomic instability?. J. Biomed. Biotechnol..

[9] Akhtar S., Najafzadeh M., Isreb M., Newton L., Gopalan R.C., Anderson D. (2020). ROS-induced oxidative damage in lymphocytes ex vivo/in vitro from healthy individuals and MGUS patients: Protection by myricetin bulk and nanoforms. Arch. Toxicol..

[10] Cemeli E., Anderson D. (2011). Mechanistic investigation of ROS-induced DNA damage by oestrogenic compounds in lymphocytes and sperm using the comet assay. Int. J. Mol. Sci..

[11] Cooke M.S., Evans M.D., Dizdaroglu M., Lunec J. (2003). Oxidative DNA damage: Mechanisms, mutation, and disease. FASEB J..

[12] Flint M.S., Baum A., Chambers W.H., Jenkins F.J. (2007). Induction of DNA damage, alteration of DNA repair and transcriptional activation by stress hormones. Psychoneuroendocrinology.

[13] Jaiswal M., LaRusso N.F., Burgart L.J., Gores G.J. (2000). Inflammatory cytokines induce DNA damage and inhibit DNA repair in cholangiocarcinoma cells by a nitric oxide-dependent mechanism. Cancer Res..

[14] Moreno-Villanueva M., Burkle A. (2016). Stress Hormone-Mediated DNA Damage Response--Implications for Cellular Senescence and Tumour Progression. Curr. Drug Targets.

[15] Rosales A.L., Cunningham J.M., Bone A.J., Green I.C., Green M.H. (2004). Repair of cytokine-induced DNA damage in cultured rat islets of Langerhans. Free Radic. Res..

[16] Thomas M., Palombo P., Schuhmacher T., von Scheven G., Bazylianska V., Salzwedel J., Schafer N., Burkle A., Moreno-Villanueva M. (2018). Impaired PARP activity in response to the beta-adrenergic receptor agonist isoproterenol. Toxicol. In Vitr..

[17] Topalovic D., Dekanski D., Spremo-Potparevic B., Djelic N., Bajic V., Zivkovic L. (2018). Assessment of adrenaline-induced DNA damage in whole blood cells with the comet assay. Arh. Hig. Rada. Toksikol..

[18] Pagel J.I., Chouker A. (2016). Effects of isolation and confinement on humans-implications for manned space explorations. J. Appl. Physiol..

[19] Smith K.J., Gavey S., NE R.I., Kontari P., Victor C. (2020). The association between loneliness, social isolation and inflammation: A systematic review and meta-analysis. Neurosci. Biobehav. Rev..

[20] Strewe C., Muckenthaler F., Feuerecker M., Yi B., Rykova M., Kaufmann I., Nichiporuk I., Vassilieva G., Horl M., Matzel S. (2015). Functional changes in neutrophils and psychoneuroendocrine responses during 105 days of confinement. J. Appl. Physiol..

[21] Basner M., Dinges D.F., Mollicone D.J., Savelev I., Ecker A.J., Di Antonio A., Jones C.W., Hyder E.C., Kan K., Morukov B.V. (2014). Psychological and behavioral changes during confinement in a 520-day simulated interplanetary mission to mars. PLoS ONE.

[22] Jacubowski A., Abeln V., Vogt T., Yi B., Chouker A., Fomina E., Struder H.K., Schneider S. (2015). The impact of long-term confinement and exercise on central and peripheral stress markers. Physiol. Behav..

[23] Yi B., Matzel S., Feuerecker M., Horl M., Ladinig C., Abeln V., Chouker A., Schneider S. (2015). The impact of chronic stress burden of 520-d isolation and confinement on the physiological response to subsequent acute stress challenge. Behav. Brain Res..

[24] Yi B., Rykova M., Feuerecker M., Jager B., Ladinig C., Basner M., Horl M., Matzel S., Kaufmann I., Strewe C. (2014). 520-d Isolation and confinement simulating a flight to Mars reveals heightened immune responses and alterations of leukocyte phenotype. Brain Behav. Immun..

[25] Shearer W.T., Lee B.N., Cron S.G., Rosenblatt H.M., Smith E.O., Lugg D.J., Nickolls P.M., Sharp R.M., Rollings K., Reuben J.M. (2002). Suppression of human anti-inflammatory plasma cytokines IL-10 and IL-1RA with elevation of proinflammatory cytokine IFN-gamma during the isolation of the Antarctic winter. J. Allergy Clin. Immunol..

[26] Smith S.M., Davis-Street J.E., Fesperman J.V., Smith M.D., Rice B.L., Zwart S.R. (2004). Nutritional status changes in humans during a 14-day saturation dive: The NASA Extreme Environment Mission Operations V project. J. Nutr..

[27] Zwart S.R., Jessup J.M., Ji J., Smith S.M. (2012). Saturation diving alters folate status and biomarkers of DNA damage and repair. PLoS ONE.

[28] Zwart S.R., Kala G., Smith S.M. (2009). Body iron stores and oxidative damage in humans increased during and after a 10- to 12-day undersea dive. J. Nutr..

[29] Hallingberg B., Turley R., Segrott J., Wight D., Craig P., Moore L., Murphy S., Robling M., Simpson S.A., Moore G. (2018). Exploratory studies to decide whether and how to proceed with full-scale evaluations of public health interventions: A systematic review of guidance. Pilot Feasibility Stud..

[30] Campos A.C., Fogaca M.V., Aguiar D.C., Guimaraes F.S. (2013). Animal models of anxiety disorders and stress. Braz. J. Psychiatry.

[31] Nagel Z.D., Chaim I.A., Samson L.D. (2014). Inter-individual variation in DNA repair capacity: A need for multi-pathway functional assays to promote translational DNA repair research. DNA Repair.

[32] Davis J.R., Fogarty J.A., Richard E.E. (2008). Human health and performance risk management—An approach for exploration missions. Acta Astronaut..

[33] Geuna S., Brunelli F., Perino M.A. (1996). Stressors, stress, and stress consequences during long-duration manned space missions: A descriptive model. Acta Astronaut..

[34] Morphew M.E. (2001). Psychological and human factors in long duration spaceflight. McGill J. Med..

[35] Vanhove A.J., Herian M.N., Harms P.D., Luthans F. (2015). Resilience and Growth in Long-Duration Isolated, Confined and Extreme (ICE) Missions.

[36] NASA (2019). Human Research Program Human Research Analog (HERA) Facility and Capabilities Information.

[37] Douglas G.L., Crucian B.E., Lorenzi H., Smith S.M., Stowe R.P., Young M.H., Zwart S.R. The Integrated Impact of Diet on Human Immune Response, the Gut Microbiota, and Nutritional Status during Adaptation to Spaceflight. https://humanresearchroadmap.nasa.gov/tasks/task.aspx?i=2068.

[38] Fenech M., Raffaele C., Martinez A., Kohlmeier M. (2020). Principles of Nutrigenetics and Nutrigenomics Fundamentals of Individualized Nutrition. Chapter 4—The Role of Nutrition in DNA Replication, DNA Damage Prevention and DNA Repair.

[39] Fenech M.F. (2010). Dietary reference values of individual micronutrients and nutriomes for genome damage prevention: Current status and a road map to the future. Am. J. Clin. Nutr..

[40] Caple F., Williams E.A., Spiers A., Tyson J., Burtle B., Daly A.K., Mathers J.C., Hesketh J.E. (2010). Inter-individual variation in DNA damage and base excision repair in young, healthy non-smokers: Effects of dietary supplementation and genotype. Br. J. Nutr..

[41] Mohrenweiser H.W., Xi T., Vazquez-Matias J., Jones I.M. (2002). Identification of 127 amino acid substitution variants in screening 37 DNA repair genes in humans. Cancer Epidemiol. Biomark. Prev..

[42] Shen M.R., Jones I.M., Mohrenweiser H. (1998). Nonconservative amino acid substitution variants exist at polymorphic frequency in DNA repair genes in healthy humans. Cancer Res..

[43] Wilson D.M., Kim D., Berquist B.R., Sigurdson A.J. (2011). Variation in base excision repair capacity. Mutat. Res..

[44] Santa-Gonzalez G.A., Gomez-Molina A., Arcos-Burgos M., Meyer J.N., Camargo M. (2016). Distinctive adaptive response to repeated exposure to hydrogen peroxide associated with upregulation of DNA repair genes and cell cycle arrest. Redox Biol..

[45] Clementi E., Inglin L., Beebe E., Gsell C., Garajova Z., Markkanen E. (2020). Persistent DNA damage triggers activation of the integrated stress response to promote cell survival under nutrient restriction. BMC Biol..

[46] Moreno-Villanueva M., Eltze T., Dressler D., Bernhardt J., Hirsch C., Wick P., von Scheven G., Lex K., Burkle A. (2011). The automated FADU-assay, a potential high-throughput in vitro method for early screening of DNA breakage. ALTEX.

[47] Moreno-Villanueva M., Pfeiffer R., Sindlinger T., Leake A., Muller M., Kirkwood T.B., Burkle A. (2009). A modified and automated version of the ’Fluorimetric Detection of Alkaline DNA Unwinding’ method to quantify formation and repair of DNA strand breaks. BMC Biotechnol..

[48] Newson R.B. (2010). Frequentist q-values for multiple-test procedures. Stata J..

