# Supplementary material for: DNA Damage and Radiosensitivity in Blood Cells from Subjects Undergoing 45 Days of Isolation and Confinement: An Explorative Study
Source: Curr Issues Mol Biol. 2022 Jan 27;44(2):654–69. doi: 10.3390/cimb44020046 (PMC8929106; doi:10.3390/cimb44020046)
Supplement: Supplementary file 1 [file cimb-44-00046-s001.zip › cimb-1506769-supplementary.pdf]

current issues in molecular biology

MDPI

Table S1. Percent reduction in median DNAI after irradiation relative to reduction at baseline, along with 95% confidence intervals and adjusted p values. # denotes adj p < 0.1.

Mission	Mission day	% Reduction DNAI	95% conf		Adjusted p-value
**3**	Day 3	9.344	-2.206	19.589	1.00E+00
**3**	Day 10	16.439	4.764	26.683	1.39E-01
**3**	Day 17	7.907	0.155	15.057	6.40E-01
**3**	Day 24	3.638	-5.137	11.680	1.00E+00
**3**	Day 31	12.029	2.174	20.890	3.20E-01
**3**	Day 38	6.374	-4.278	15.937	1.00E+00
3	**Day 45**	**14.545**	**6.151**	**22.188**	**2.82E-02 #**
**3**	R+1	10.157	2.303	17.379	2.25E-01
3	**R+7**	**17.605**	**10.199**	**24.401**	**4.35E-04 #**
**5**	Day 3	1.226	-6.120	8.065	1.00E+00
5	**Day 10**	**19.959**	**14.769**	**24.833**	**4.44E-10 #**
5	**Day 17**	**18.991**	**13.834**	**23.838**	**1.88E-09 #**
5	**Day 24**	**22.882**	**13.703**	**31.084**	**2.68E-04 #**
5	**Day 31**	**17.856**	**11.136**	**24.067**	**5.05E-05 #**
**5**	Day 38	0.869	-4.792	6.225	1.00E+00
**5**	Day 45	2.642	-6.567	11.056	1.00E+00
**5**	R+1	6.131	-2.321	13.884	1.00E+00
**5**	R+7	4.132	-5.627	12.989	1.00E+00

Table S2. Change in mean ADRR (DNAI/hr), 95% confidence intervals, and adjusted p-values. # denotes adj p < 0.1.

Mission	Mission day	Δ ADRR	95% conf		Adjusted p-value
**1**	Day 3	-0.026	-0.104	0.053	1.0000E+00
**1**	Day 10	0.033	-0.049	0.115	1.0000E+00
**1**	Day 17	0.017	-0.036	0.070	1.0000E+00
**1**	Day 24	0.071	0.009	0.133	3.8145E-01
**1**	Day 38	0.057	0.008	0.107	3,8145E-01
1	**Day 45**	**0.088**	**0.038**	**0.137**	**1.6092E-02 #**
**1**	R+1	0.043	-0.011	0.098	1.0000E+00
**1**	R+7	0.034	-0.016	0.084	1.0000E+00
**2**	Day 3	0.020	-0.029	0.068	1.0000E+00
**2**	Day 10	0.050	0.014	0.086	1.3926E-01
2	**Day 17**	**0.056**	**0.020**	**0.091**	**5.4293E-02 #**
**3**	Day 3	-0.067	-0.188	0.055	1.0000E+00
**3**	Day 10	-0.106	-0.196	-0.017	3.3523E-01
**3**	Day 17	-0.017	-0.118	0.084	1.0000E+00
3	**Day 24**	**-0.173**	**-0.246**	**-0.100**	**1.5742E-04 #**
**3**	Day 31	-0.030	-0.134	0.073	1.0000E+00
**3**	Day 38	-0.048	-0.116	0.020	1.0000E+00
**3**	Day 45	-0.066	-0.165	0.033	1.0000E+00
3	**R+1**	**-0.152**	**-0.212**	**-0.093**	**2.9080E-05 #**
**3**	R+7	0.012	-0.080	0.103	1.0000E+00
4	**Day 3**	**-0.038**	**-0.061**	**-0.015**	**3.0595E-02 #**
**4**	Day 10	0.001	-0.037	0.038	1.0000E+00
**4**	Day 17	-0.019	-0.048	0.010	1.0000E+00
**4**	Day 24	-0.036	-0.073	0.000	6.8533E-01
4	**Day 31**	**-0.035**	**-0.057**	**-0.014**	**3.1416E-02 #**
4	**Day 38**	**-0.083**	**-0.109**	**-0.058**	**1.2030E-08 #**
4	**Day 45**	**-0.085**	**-0.142**	**-0.028**	**7.2355E-02**
**4**	R+7	-0.033	-0.066	0.000	6.9458E-01
5	**Day 3**	**0.049**	**0.018**	**0.080**	**4.3669E-02 #**
5	**Day 10**	**0.106**	**0.079**	**0.134**	**8.8390E-12 #**
5	**Day 17**	**0.209**	**0.171**	**0.247**	**2.8000E-24 #**
5	**Day 24**	**0.099**	**0.049**	**0.148**	**3.5439E-03 #**
5	**Day 31**	**0.086**	**0.031**	**0.141**	**5.0631E-02 #**
5	**Day 38**	**0.083**	**0.034**	**0.131**	**2.3134E-02 #**
5	**Day 45**	**0.162**	**0.115**	**0.209**	**1.2100E-09 #**
5	**R+1**	**0.197**	**0.161**	**0.233**	**3.0690E-24 #**
**5**	R+7	0.022	-0.002	0.045	8.8174E-01

Table S3. Allocation of samples to 7 experimental conditions for a given subject and mission time point as originally planned.

Exp Condition	Radiation	Incubation Time (min)	Replicates
**1**	pre-irradiation	none	4
**2**	3.73 Gy	0	4
**3**	3.73 Gy	10	4
**4**	3.73 Gy	20	4
**5**	3.73 Gy	30	4
**6**	3.73 Gy	40	4
**7**	3.73 Gy	50	4
			Total = 28
